# Extensive Molecular Dynamics Simulations Disclosed the Stability of mPGES‐1 Enzyme and the Structural Role of Glutathione (GSH) Cofactor

**DOI:** 10.1002/minf.202200140

**Published:** 2022-09-29

**Authors:** Simone Di Micco, Gianluigi Lauro, Giuseppe Bifulco

**Affiliations:** ^1^ European Biomedical Research Institute of Salerno (EBRIS) via Salvatore De Renzi 50 84125 Salerno Italy; ^2^ Dipartimento di Farmacia University degli Studi di Salerno, via Giovanni Paolo II 132 84084 Fisciano (SA) Italy

**Keywords:** mPGES-1, molecular dynamics, MM-GBSA, cancer, inflammation

## Abstract

A deep in silico investigation of various microsomal prostaglandin E_2_ synthase‐1 (mPGES‐1) protein systems is here reported using molecular dynamics (MD) simulations. Firstly, eight different proteins models (Models A−H) were built, starting from the active enzyme trimer system (Model A), namely that bound to three glutathione (GSH) cofactor molecules, and then gradually removing the GSHs (Models B−H), simulating each of them for 100 ns in explicit solvent. The analysis of the obtained data disclosed the structural role of GSH in the chemical architecture of mPGES‐1 enzyme, thus suggesting the unlikely displacement of this cofactor, in accordance with experimentally determined protein structures co‐complexed with small molecule inhibitors. Afterwards, Model A was submitted to microsecond‐scale molecular dynamics simulation (total simulation time=10 μs), in order to shed light about the dynamical behaviour of this enzyme at atomic level and to obtain further structural features and protein function information. We confirmed the structural stability of the enzyme machinery, observing a conformational rigidity of the protein, with a backbone RMSD of ∼3 Å along the simulation time, and highlighting the strong active contribution of GSH molecules due to their active role in packing the protein chains through a tight binding at monomer interfaces. Furthermore, the focused analysis on R73 residue disclosed its role in solvent exchange events, probably excluding its function as route for GSH to enter towards the endoplasmic reticulum membrane, in line with the recently reported function of cap domain residues F44‐D66 as gatekeeper for GSH entrance into catalytic site.

## Graphical abstract

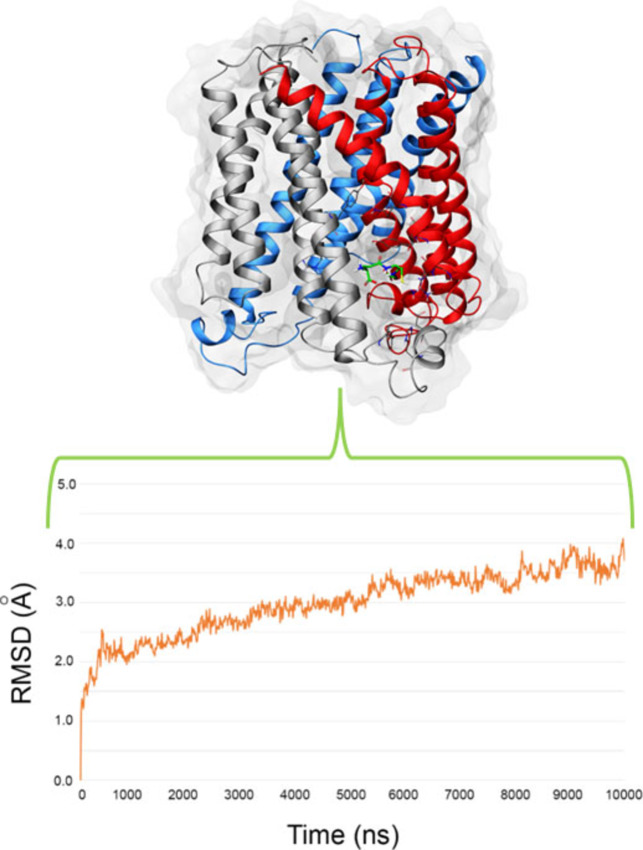

## 1 Introduction

Prostaglandins (PGs) are physiologically active lipid compounds accomplishing several functions. They are formed from free arachidonic acid (AA) by cyclooxygenases (COXs) together with specific terminal PG synthases.^[1]^ Among the different PGs, the prostaglandin E_2_ (PGE_2_) represents a key effector of inflammation and cancer,^[2]^ regulating cell apoptosis, proliferation, angiogenesis, and immune surveillance.^[3]^ Interestingly, high levels of PGE_2_ have been detected in human colon adenomas and in adenocarcinomas.^[4]^ Microsomal prostaglandin E_2_ synthase‐1 (mPGES‐1) is the key enzyme responsible of its biosynthesis, since it catalyzes the conversion of prostaglandin H_2_ (PGH_2_) to PGE_2_, assisted by the cofactor glutathione (GSH).^[5]^ Differently from mPGES‐2 and cPGES as constitutively expressed protein family members, mPGES‐1 enzyme represents the inducible membrane‐bound isoform. Its expression increases in response to pro‐inflammatory stimuli and it is involved in several acute and chronic disorders,^[6]^ such as arthritis and rheumatoid arthritis, pain, inflammation and cancer.[^7^, ^8^] For these reasons, mPGES‐1 has been recognized as a promising target for anti‐inflammatory and anticancer therapies as well as representing a valuable alternative in the treatment of chronic inflammation‐related disorders.^[9]^ Indeed, mPGES‐1 suppression reduces the typical side effects of the COX‐2 inhibitors and, accordingly, this strategy could be useful for disclosing new promising and safer drugs blocking the inflammation‐induced biosynthesis of PGE_2._ ^[10]^ Different mPGES‐1 inhibitors were reported featuring a high chemical variability, while only two inhibitors entered clinical development phases, specifically: GRC 27864 (Glenmark Pharmaceuticals Ltd) and LY3023703 (developed by Eli Lilly), the latter unfortunately showing liver toxicity that caused the interruption of the related approval process.^[11]^

**Figure 1:**
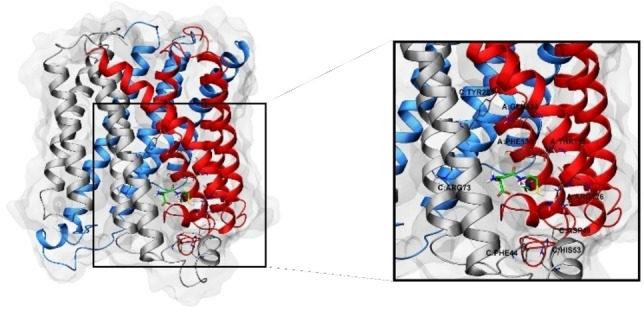
mPGES‐1 structure. On the left: mPGES‐1 tertiary structure (chain A red, chain B light blue, chain C light grey, molecular surface represented in transparent light grey); on the right, focused representation of the mPGES‐1 ligand binding site; glutathione (GSH) cofactor and key‐residues in the mPGES‐1 binding site are depicted in sticks (C: green for glutathione and grey for the other residues, O red, N, blue, polar H light gray, S yellow).

The careful analysis at molecular level, mainly driven by crystallographic studies, disclosed mPGES‐1 as a homotrimer with three identical active sites each containing a GSH molecule. Each monomer presents four alpha helixes spanning through the membrane of the endoplasmic reticulum (Figure 1). In particular, different high resolution X‐ray structures of the enzyme also co‐complexed with high‐affinity inhibitors[^12^, ^13^] revealed important structural details. X‐ray models highlighted an observable kink in helix II giving rise to a large cytoplasmic cone‐shaped cavity in the center of the mPGES‐1 trimer. The central cavity is adjacent to the three active sites containing the GSH cofactors. Moreover, two conformations for R73 side chain were found; in the first one, the R73 residue interacted with a carboxylate group of GSH, preventing the connection with the cone‐shaped cavity whereas, in the second conformation, the R73 was close to the backbone carbonyl of L69 opening the access to the cone central cavity. The role of the interconnected super pocket is still unclear. Several hydrogen bonds into transmembrane helixes confer a conformational stability to mPGES‐1, despite the generally observed conformational flexibility of membrane proteins. Furthermore, the comparison of X‐ray structures of mPGES‐1 bound to GSH (PDB ID: 4AL0)^[12]^ and its analogue (PDB code: 4AL1),^[12]^ presenting a bulky biphenyl substituent, highlighted no significant conformational changes except a slight shift of Y130 residue. Also, the superposition of further mPGES‐1 structures co‐complexed with small molecules confirmed this outcome.[^13^, ^14^, ^15^]

The catalysis mechanism exerted by mPGES‐1 was investigated during the last few years, based on the atomistic details arising from the X‐ray structures and on the reported mutagenesis data and, in the regard, a 1 : 3‐site reactivity by mPGES‐1 was originally proposed.^[16]^ Also, when the first high‐resolution X‐ray structure of human mPGES‐1 was released, S127 residue was identified as hydrogen bond donor able to stabilize thiolate anion formation within the GSH cofactor. Afterwards, site‐directed mutagenesis and activity assays highlighted that this residue is actually not required for mPGES‐1 activity. On the other hand, a key and dynamic interaction between D49 and R126 together with a crystallographic water molecule was recognized as essential for elucidating the catalysis mechanism.

Starting from these considerations and in order to shed light about the structural features of mPGES‐1 by a dynamic point of view, the following aspects were investigated in this study:

monitoring the structural role of GSH in mPGES‐1 protein architecture. With this aim, molecular dynamics simulations were performed accounting distinct protein systems differing from the presence/absence and the total number of GSH cofactor molecules. This information is beneficial to clarify the structural role of the cofactor in the mPGES‐1 machinery;
evaluating the conformational stability of the native mPGES‐1 protein structure, namely bound to three GSH molecules. In particular, extensive molecular dynamics simulations were performed in the microsecond scale to assess the stability of the protein architecture and to monitor the dynamic interactions established by GSH molecules and the protein counterparts;
elucidating the role of R73 as gatekeeper between active site and cone‐shaped cavity, and if the connected pockets are responsible for the solvent exchange or as entrance of GSH into endoplasmic reticulum membrane. This can give new insights on the protein machinery and can suggest the cone‐shaped cavity as another binding region to modulate mPGES‐1 catalytic activity by small molecules.

## 2 Computational Methods

### 2.1 Molecular Dynamics Simulations

Firstly, the X‐ray structure of apo mPGES‐1 (PDB ID: 4AL0)^[12]^ was processed with Protein Preparation Wizard,^[17]^ from Schrödinger suite: all hydrogen addition; bond order assignment; checking of residue alternate position; checking of missing side chains and loops. The charges of amino acid side chains were assigned considering their p*K*_a_ at pH 7.0. The H‐bond network was refined through the “optimize” option of Protein Preparation Wizard. The so obtained Model A was used to build the Models B–H by removing the related GSH molecules (Table 1). The Models A–H were prepared for simulations by means of System Builder^[18]^ in Desmond.[^19^, ^20^] An orthorhombic box was built with a 10 Å buffer distance, resulting in a system with approximately 86657 atoms. The TIP3P^[21]^ water model for solvation, POPC as model membrane, and OPLS‐2005 force field were used,^[22]^ and Na^+^ and Cl^−^ ions were added for electroneutrality. An additional NaCl solution (0.15 M) was applied. The Models A–H systems were equilibrated by the following relaxation protocol: 1) minimization with restrained solute heavy atoms (50 kcal/mol), by LBFGS method, 2000 iterations, convergence threshold of 50.0 kcal/mol/Å; 2) unrestrained minimization by LBFGS method, 2000 iterations, convergence threshold of 5.0 kcal/mol/Å; 3) 0.3 ns of NVT simulation at 310 K, with restrained solute heavy atoms (50 kcal/mol); 4) 1 ns of NPT simulation (310 K) with restrained solute heavy atoms (10 kcal/mol) and H_2_O barrier; 5) 0.5 ns of NPT simulation (310 K) of solvent and lipids with restrained solute heavy atoms (10 kcal/mol); 6) 3 ns of NPT simulation (310 K) with restrained solute heavy atoms (10 kcal/mol); 7) 0.5 ns of NPT simulation (310 K) with restrained Cα protein atoms (2 kcal/mol); 8) unrestrained 5 ns of NPT simulation (310 K). Each step of equilibration protocol was checked by Simulation Quality Analysis tool of Desmond, monitoring the total energy, potential energy, temperature, pressure and volume.

**Table 1:** mPGES‐1 models used in molecular dynamics simulations.

Name	description
Model A	mPGES‐1 bound to three GSH molecules
Model B	mPGES‐1 without GSH_A_
Model C	mPGES‐1 without GSH_B_
Model D	mPGES‐1 without GSH_C_
Model E	mPGES‐1 without GSH_AB_
Model F	mPGES‐1 without GSH_BC_
Model G	mPGES‐1 without GSH_AC_
Model H	mPGES‐1 without GSH_ABC_

In order to assess the biophysical validity of the built systems, the average area per lipid headgroup (APL) and bilayer thickness measurements for each built system was measured using Grid‐MAT‐MD.^[23]^ The corresponding averaged area per lipid headgroup of the extracellular leaflet (eAPL) and of the intracellular leaflet (iAPL) in the first nanosecond (eAPL1 ns and iAPL1 ns) and in the last nanosecond (eAPL9 ns and iAPL9 ns) of the equilibration for all the considered complexes is reported in Table S1. The calculated values are in agreement with the experimental values measured for 1‐palmitoyl‐2‐oleoyl‐sn‐glycero‐3‐phosphocholine (POPC) lipid bilayers.^[24]^ Bilayer system representation and the performed thickness analysis, for each built system at the end of the equilibration phase, are reported in Table S1. MD simulations (310 K) were carried out with: a recording interval of 1.2 ps; an ensemble class NPT (1.01 bar); 2.0 fs integration timestep. The statistical significance of Model A–H simulation was assessed by a representative replica of Model H obtaining an averaged RMSD of 0.183 Å (Figure S1).

The equilibrated system (see above) was underwent to metadynamics simulation (50 ns, 310 K), by setting two collective variables (CVs) and using defalut parameters of Desmond:[^19^, ^20^] Gaussians were deposited every 0.09 ps with a starting height of 0.03 kcal/mol. CV1 is Χ_3_ dihedral angle (width=5°). CV2 is the distance of centres of mass of GSH and binding site residues (Table 2), setting the Gaussian width=0.05 Å and a Wall=15 Å.

**Table 2:** MM‐GBSA Interaction Energies (±SEM, kcal/mol) for amino acids around 5 Å from GSH. The values in bold are ≤−1 kcal/mol.

residue	GSH
A31	**−1.76±**0.03
T34	**−2.08±**0.04
G35	−0.35±0.01
R38	**−4.58±**0.15
N46	−0.07±0.02
L69	−0.82±0.02
R70	−0.91±0.02
H72	−0.54±0.38
R73	**−6.93±**0.13
N74	**−5.44±**0.42
E77	0.71±0.09
H113	**−3.76±**0.06
Y117	**−1.82±**0.06
R126	**−3.78±**0.09
S127	**−1.19±**0.41
Y130	**−3.43±**0.12

The essential dynamics analysis was performed by ProDy.[^25^, ^26^, ^27^]

### 2.2 MM‐GBSA

MM‐GBSA calculations were carried out on frame at time**=**0 ns of Model A by the Prime^[28]^ module of the Schrödinger suite by applying default parameters, keeping the macromolecule rigid. The implicit membrane option was used, placing the membrane according to the information stored in the OPM database file. The binding energy was calculated for each glutathione in presence or absence of the other two glutathione molecules. The obtained values were averaged by three glutathione molecules (A‐C) and reported with corresponding SEM. Alanine scanning was performed, modifying one by one the binding site residues and calculating the relative binding energy, which was averaged by three protein subunits (± SEM).

## 3 Result and Discussion

### 3.1 Investigating the Structural Role of GSH

In order to assess the structural role of GSH cofactor in mPGES‐1, eight models (Models A–H) were built (Table 1) from highly resolved X‐ray structure of mPGES‐1 (PDB ID: 4AL0).

In details, we considered the mPGES‐1 trimer accommodating a GSH molecule for each catalytic site (Model A). Models B–D were generated by removing the GSH from chain A, chain B and chain C, respectively. The Models E–G were built removing two GSH molecules: from chains A and B, from chains C and B, from chain C and A, respectively. Model H did not feature GSH molecules. The obtained models were investigated by means of unrestrained molecular dynamics[^29^, ^30^] (temperature=310 K, simulation time=100 ns) with explicit solvent water (TIP3P) and membrane (POPC) models.

**Figure 2:**
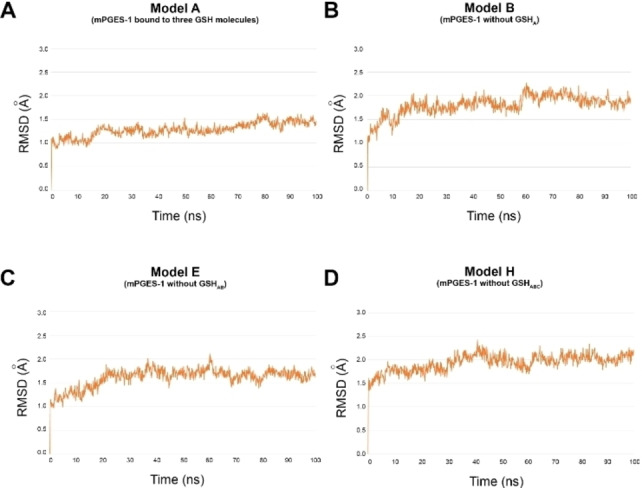
RMSD (Å) plots for Model A (panel A), B (panel B), E (panel C), and H (panel D) systems, related to mPGES‐1 enzyme differently bound to GSH molecules (see Table 1), computed from molecular dynamics simulations (simulation time=100 ns).

The results of the MD simulations (100 ns, 310 K) highlighted that Model A presented a great stability compared to the other investigated Models (B–H), with an atom‐positional RMSD for the backbone <1.6 Å (Figure 2). On the contrary, Model G showed larger fluctuations during the molecular dynamics trajectory, giving rise to 1 Å deviation of RMSD value at 47 ns (Figure S2). The Model G lacks GSHs from chains A and C, both connected to monomer A in Model A, justifying the largest atom movement of the chain. Interestingly, for Model H, without GSH molecules, we observed lower RMSD of chains compared to Model G. This could be ascribed to the balanced atomic movements of each single chain, which leads to a higher flexibility for the absence of bound GSH molecules. In the remaining molecular systems (Models B–F), the chain A is bound at least with one GSH molecule, giving rise to lower fluctuations respect to Model G, with a trajectory trend similar to the Model A. However, we observed in Model C (namely in the absence of GSH_B_, not directly in contact with chain A) induced fluctuations of the polypeptide chain, due to the larger atomic movements of other monomers lacking GSH contacts.

Similar considerations could be made for chain B. Larger fluctuations were observed for Models presenting monomer B without contacts with GSH from chains A and B (Models B, C, E, Figures 2, S2). Even in the absence of GSH from chain C, not establishing direct interactions with chain B, some fluctuations were detected. As for chain A, chain B in Model H showed deviations with respect to the Model A, in which mPGES‐1 was bound to all the three GSH molecules.

As expected, comparable outcomes were obtained for the analysis of chain C. In the Model A, the chain C showed the highest stability, whereas for the Model H the largest RMSD deviations were noticed due to the absence of the cofactors. As for chains A and B, models without a GSH bound to chain C presented greater RMSD values. However, the absence of GSH_A_ causes some indirect fluctuations on the chain C.

The analysis of the whole trimer is comparable to the outcomes from single chains, highlighting the structural stabilizing role of GSHs. Specifically, the removal of GSH resulted into a lower packing of enzyme monomers. The lowest RMSD values were observed in Model A, whereas increasing fluctuations were detected for Model H. It is noteworthy that the Model G, devoid of GSH from chains A and C, gave rise to the larger atomic fluctuations, particularly at 50 ns of simulation.

**Figure 3:**
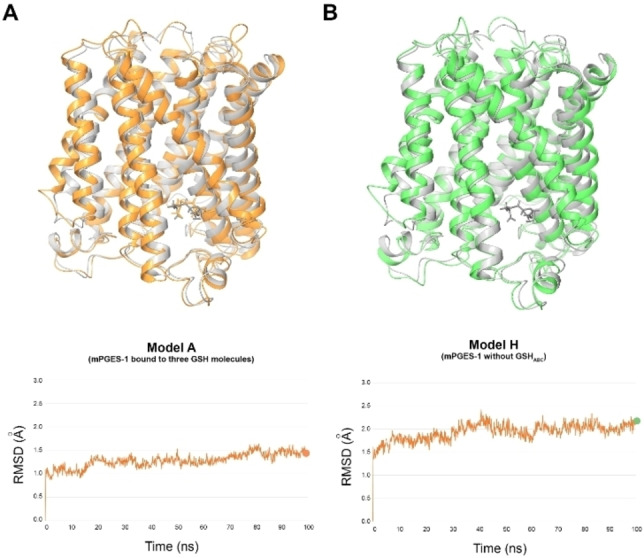
Superposition between mPGES‐1 crystal structure (PDB code: 4AL0) (represented in light grey ribbons) and the related three‐dimensional structure obtained after molecular dynamics simulation at 100 ns for A) Model A (represented in orange ribbons) and B) Model H (represented in green ribbons). The RMSD (Å) values between the superimposed structures are highlighted on the Model A and Model H RMSD plots (bottom) with orange and green points, respectively.

By comparing Models A and H (Figure 3), considerable structural differences were observed around the catalytic site. In particular, the superposition of the mPGES‐1 conformations at the end of the molecular dynamics rounds (100 ns) for models A and H, with respect to X‐ray structure (PDB ID: 4AL0), highlighted in the latter case a structural shift of the helix I and of the cap domain, which could induce new and larger shape into the catalytic site and eventually favour the entrance to the enzyme pocket (Figure S3). Indeed, for Model A we observed just side chain fluctuations over time. It is worth of note that the movement of cap domain was in agreement with the data reported by Zhou et al.^[31]^

### 3.2 Microsecond‐scale Molecular Dynamics Simulation of mPGES‐1 Bound to Three GSHs (Model A)

**Figure 4:**
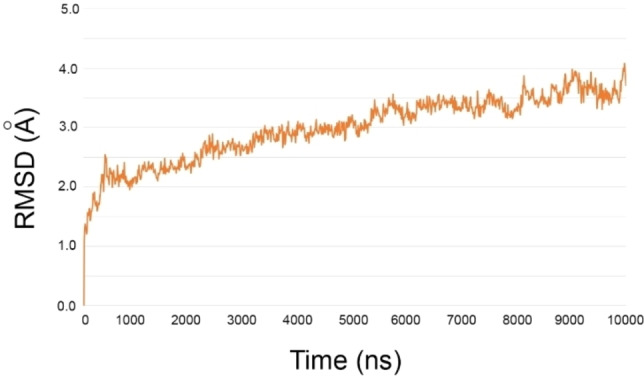
The backbone RMSD as function of simulation time (ns) of the trimer over simulation time.

In the previous section, we investigated and confirmed the putative structural role of GSH as stabilizing the enzyme conformation. Based on these outcomes, we further explored the structural features by microsecond‐scale simulation of the whole trimer bound to three GSH molecules (Model A, Table 1). Globally, we observed a stable trajectory during the entire simulation, suggesting that Model A is endowed of an inherent conformational rigidity (Figure 4).

Even at very long simulation time, the GSH‐mPGES‐1 molecular system showed expected atom fluctuations, especially by the loops. In particular, the 54–62 residues of C‐domain, which does not present any direct contact with GSH, gave rise to larger oscillations then the remaining enzyme portions. The fluctuation of the portion affects spatial position of helix I at 5.1 μs (Figure S4). These observations were also corrobarated by essential dynamics analysis (Figure S5), suggesting the movement of 54–62 residues of C‐domain as the most distinctive.

The careful analysis of the MD simulation disclosed that the observed limited flexibility is mainly due to several hydrogen bonds into transmembrane helixes conferring a conformational stability to mPGES‐1, despite the generally observed conformational flexibility of membrane proteins, such as transporters^[32]^ and G protein‐coupled receptors.^[33]^ In a recent contribution,^[14]^ we reported the comparison of the mPGES‐1 X‐ray model (PDB ID: 4AL0) with structures of the enzyme bound to different inhibitors. From this analysis, we observed a backbone RMSD ranging from 0.066 Å to 0.419 Å, without any protein conformational rearrangements upon ligand binding, supporting the detected structural rigidity of mPGES‐1. The tight binding of GSH molecules further contributes to the conformational stability of the enzyme, as also revealed by ΔG_bind_=‐67.35±0.99 kcal/mol (calculated by MM‐GBSA methodology) averaged for three cofactor molecules on Model A (at time=0 ns). This binding energy value was calculated considering the other two glutathione binding sites in the free state. Interestingly, the binding energies values of cofactor was not affected by the presence of other two glutathione molecules: ΔG_bind_=‐67.86±0.09 kcal/mol. Specifically, each GSH molecule binds mPGES‐1 at interface of two monomers widening their contacts and increasing the packing of protein chains. The breakdown of MM‐GBSA binding free energy, accounting the residues surrounding (5 Å) GSH (Table 2), was investigated to distinguish the amino acids highly contributing to the small molecule interactions.[^34^, ^35^] The residues, showing a ΔG ≤−1 kcal/mol, were considered as hotspots.[^34^, ^35^]

Our analysis showed that amino acids A31, T34, R38, R73, N74, H113, Y117, R126, S127, and Y130 gave values <‐1 kcal/mol, significantly contributing to the ligand binding than the other analysed residues. In particular, it is worth of note that R38, R73 and N74 presented a ΔG_bind_ <−4 kcal/mol. Basically, both R38 and R73 established a salt bridge and two hydrogen bonds with carboxylate groups of glutathione (Figure S6). The N74 was hydrogen bonded with terminal carboxylate of glutathione glycine and with side chain carbonyl of GSH glutamate. The residues H113, Y117, R126, and S127 were involved in a single H‐bond, whereas A31, T34, and Y130 gave van der Waals interactions. This analysis was complemented and confirmed by an alanine scanning investigation (Table S2). Indeed, the identified hot spot residues showed a drastic reduction of ΔG_bind_ upon alanine conversion, highlighting their key role in GSH recognition. On the contrary, the other residues, suggested as non‐hotspots, gave comparable ΔG_bind_ values between wild type and alanine converted states.

**Figure 5:**
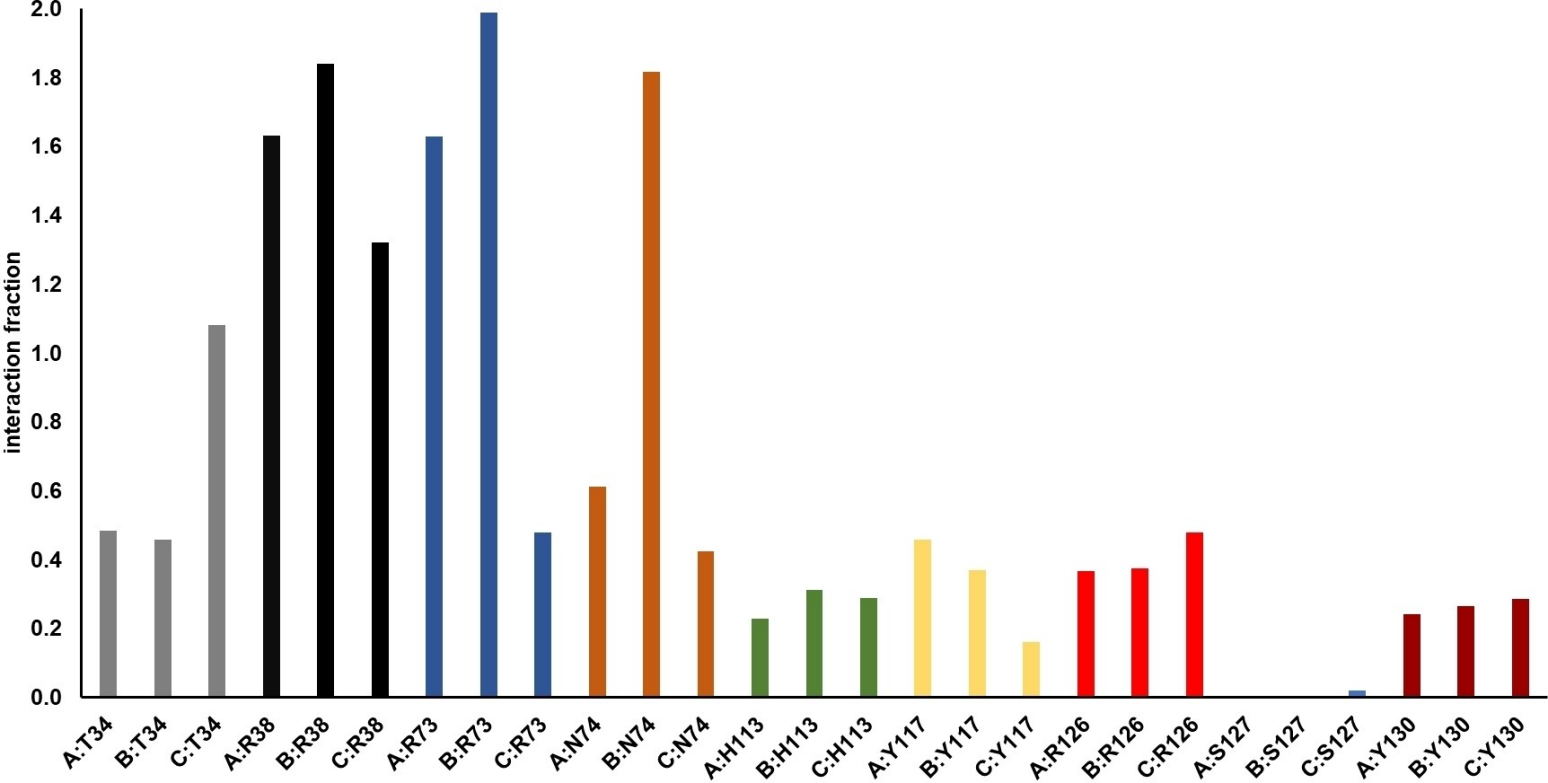
Protein‐ligand contact histograms during the simulation of identified hotspots by MM‐GBSA analysis. Protein‐ligand interactions are categorized into four types: hydrogen bonds, hydrophobic, ionic and water bridges. The stacked bar charts are normalized over the course of the trajectory: for example, a value of 0.7 suggests that 70 % of the simulation time the specific interaction is maintained. Values >1.0 indicate that some protein residue may make multiple contacts of same subtype with the ligand.

Afterwards, we evaluated the stability over time of the interactions given by GSH molecules with the identified hotspots. The analysis showed the qualitative agreement between the MM‐GBSA investigation and the trend observed by molecular dynamics simulation. Specifically, R38, R73, and N74, featuring a ΔG_bind_ <−4 kcal/mol, maintained most of the contacts with GSH (Figure 5) during the whole trajectory (>50 %).[^36^, ^37^, ^38^] The residue T34 showed a similar behaviour if compared with R38, R73, and N74 but with lower interaction fractions, in agreement with ΔG_bind_=−2.08±0.04 kcal/mol. The residues H113, Y117, R126, S127, and Y130 featured a similar profile for each chain and lower interactions fraction over time respect to the hotspots, in agreement with MM‐GBSA analysis. By inspection of monomer trend for the identified hotspots, we observed that residues from monomer C showed a different profile with respect to those belonging to chains A and B. The T34 from chain C (C:T34) showed a larger interaction fraction respect to the same residue on the other monomers. On the contrary, the C:R38 and C:R73 gave less contacts over the simulation time with respect to R38 and R73 of chains A and B. For N74, we observed that the residue belonging to monomer B established more contacts over the time than those related to chains A and C.

**Figure 6:**
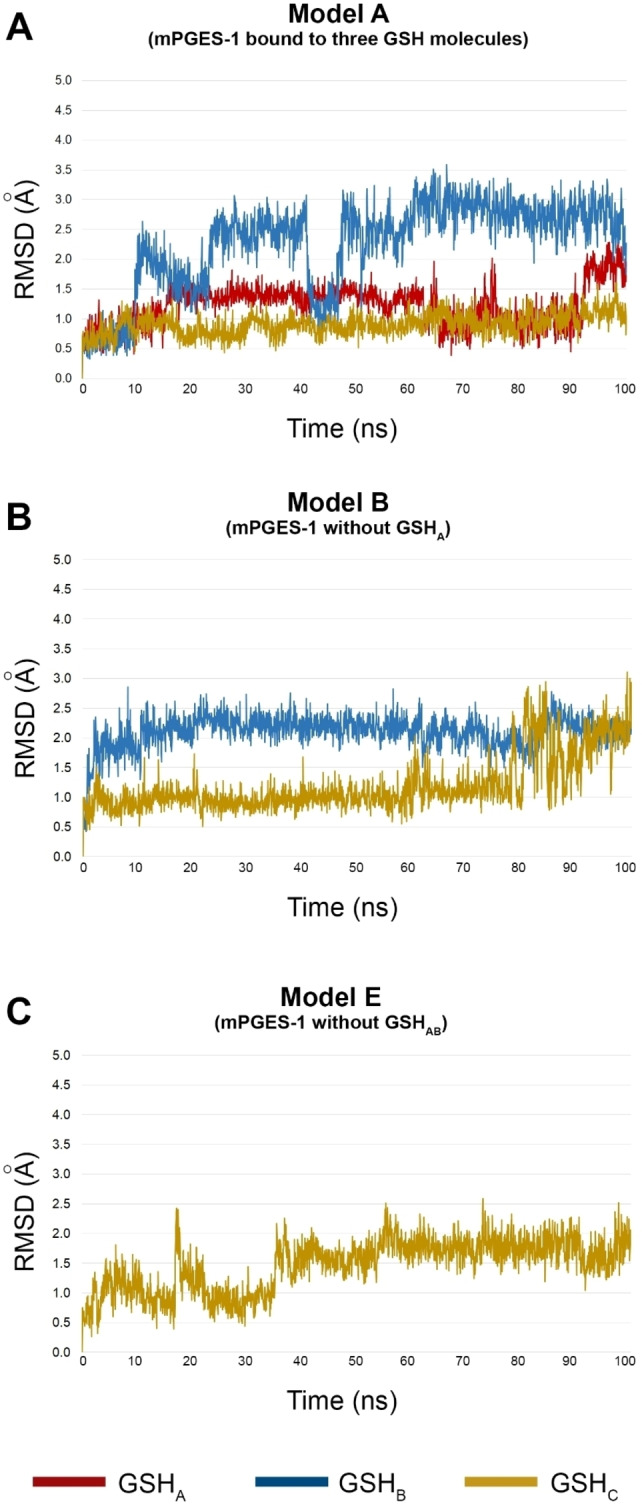
The GSH RMSD values as function of simulation time (ns).

As expected, the analysis of RMSD of GSH during the simulation matched the trajectory stability as found for the protein (Figure 6). These observed structure stability of mPGES‐1 during our simulations is an agreement with reported hypothesis of Sjögren et al., who determined the first high resolution X‐ray structure of mPGES‐1 (PDB ID: 4AL0), suggesting a structural rigidity of the enzyme from experimental model.

According to the analysis reported above with Models B–H, we observed an asymmetric behaviour of each monomer. Both chains A and C showed larger fluctuations respect to the chain B.

### 3.3 Analysis of R73

The X‐ray models revealed an observable kink in helix II for each monomer giving rise to a large cytoplasmic cone‐shaped cavity in the centre of the mPGES‐1 trimer. The central cavity is adjacent to the three active sites containing the GSH. Two distinct conformations for R73 side chain are observed in the X‐ray crystallography structure (PDB ID: 4AL0). In one conformation the R73 interacts with glycine carboxylate group of GSH, hampering the connection with the cone‐shaped cavity. In a second conformation the R73 binds the backbone carbonyl of L69, opening the access to the cone central cavity and forming an interconnected super pocket. The role of the generated larger cavity is unclear: either the connected pockets could be responsible for the solvent exchange or could allow the entrance of hydrophilic GSH into catalytic site placed inside the hydrophobic bilayer of endoplasmic reticulum membrane. To elucidate the role of R73 as gate between active site and cone‐shaped cavity, we analysed the Root Mean Square Fluctuation (RMSF) of its side chain (Figure S7).

**Figure 7:**
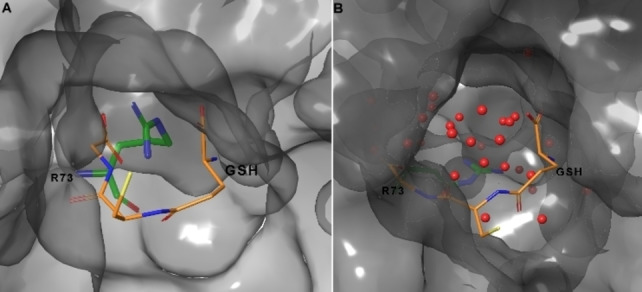
The conformational arrangements of R73 at simulation time 0 (A) and >2 μs (B). The protein is represented by grey molecular surface, whereas R73 and GSH by green and orange tubes, respectively, with the following atom color code: C, as for tubes; O, red; N, blue; S yellow. The water molecules are depicted as red balls.

From this analysis, we observed the preservation of the interaction between side chain of R73 and glycine carboxylate of GSH from all chains. Beyond 2 μs, the breakage of the interaction is induced by the conformational change of C:R73 at 2 μs. The same observations could be made for A:R73 at about 2.6 μs, whereas the B:R73 keeps the interaction during all simulation. Overall, we did not observe the exit of GSH (Figure 7), but the filling of the interconnected cavity by water molecules. Our theoretical investigation could suggest the role of the generated pocket for solvent exchange. This observation is in agreement with the recently proposed catalytic mechanism of mPGES‐1,^[39]^ which suggested a prominent role of a water molecule for the PGE_2_ production.

Moreover, our results are in line with recent outcomes reported by Zhou et al.^[31]^ on the role of cap domain region, delimited by residues F44‐D66, which switches between open and closed conformations acting as gate for GSH entrance into catalytic site. A preliminary metadynamics study (50 ns, Figure S8) was carried out to integrate microsecond‐scale molecular dynamics simulation. The metadynamics results confirmed the observations derived from long molecular dynamics simulation.

## 4 Conclusions

The investigation of the dynamical behaviour of mPGES‐1 at atomic level allowed to deepen some structural features and protein functions.

The analysis of R73 suggested that the residue is involved in solvent exchange rather than as route for GSH to enter endoplasmic reticulum membrane. The microsecond‐scale simulation was also corroborated by a preliminary metadynamics analysis. The obtained results are in line with the recently reported data regarding the role of cap domain residues F44‐D66, as gate for GSH entrance into the catalytic site. Moreover, our suggested role of R73 could support the lately proposed leading role of water molecules into PGE_2_ biosynthesis. Furthermore, these new insights on the protein machinery could suggest the cone‐shaped cavity as another binding region to modulate mPGES‐1 catalytic activity by small molecules. Indeed, new inhibitors able to bind the central cavity could be designed and, through their interactions with R73, they could block the water molecule exchange involved in the catalytic events. However, these findings deserve further studies with enhanced sampling methods, such as steered MD or metadynamics.

Monitoring the protein dynamics at microsecond scale, we observed a conformational rigidity of the protein, with a backbone RMSD of ∼3 Å along the simulation time. As expected, the reduced flexibility is ascribed to hydrophobic contacts of transmembrane helixes and interhelical hydrogen bonds of polar residues located at the interface among monomers. Our investigation also highlights the strong contribution from GSH molecules to the conformational stability of the enzyme. Specifically, GSH increased the packing of protein chains through a tight binding at monomer interface extending their interactions. Thus, our analysis would not suggest a possible allosteric modulation of the enzyme by small molecules, and further studies could be useful to deepen this aspect. This structural rigidity did not hamper the observation that a single monomer can differently fluctuate respect to the other chains, but its spatial rearrangements are sterically stabilized by the other homopolymers. These findings, along with the different fluctuations of R73 from each chain, further support the one‐third‐of‐the‐sites reactivity underlying the catalytic activity of mPGES‐1.

## Conflict of interest

None declared.

5

## Supporting information

As a service to our authors and readers, this journal provides supporting information supplied by the authors. Such materials are peer reviewed and may be re‐organized for online delivery, but are not copy‐edited or typeset. Technical support issues arising from supporting information (other than missing files) should be addressed to the authors.

Supporting Information

## Data Availability

Data sharing not applicable to this article as no datasets were generated or analysed during the current study.
