# Supplementary material for: Extensive Molecular Dynamics Simulations Disclosed the Stability of mPGES‐1 Enzyme and the Structural Role of Glutathione (GSH) Cofactor
Source: Mol Inform. 2022 Sep 29;41(12):2200140. doi: 10.1002/minf.202200140 (PMC10078397; doi:10.1002/minf.202200140)
Supplement: Supplementary file 1 — Supporting Information [file MINF-41-0-s001.pdf]

molecular informatics

Supporting Information

Extensive Molecular Dynamics Simulations Disclosed the Stability of mPGES-1 Enzyme and the Structural Role of Glutathione (GSH) Cofactor

Simone Di Micco,* Gianluigi Lauro, and Giuseppe Bifulco*© 2022 The Authors. Molecular Informatics published by Wiley-VCH GmbH;. This is an open access article under the terms of the Creative Commons Attribution License, which permits use, distribution and reproduction in any medium, provided the original work is properly cited.

Extensive molecular dynamics simulations disclosed the stability of mPGES-1 enzyme and the structural role of glutathione (GSH) cofactor

Simone Di Micco,*[a] Gianluigi Lauro,[b] and Giuseppe Bifulco*[b]

[a] European Biomedical Research Institute of Salerno (EBRIS),
via Salvatore De Renzi 50,
84125 Salerno, Italy
[b] Dipartimento di Farmacia, University degli Studi di Salerno,
84084 Fisciano (SA), Italy
*e-mail: s.dimicco@ebris.eu (SDM); bifulco@unisa.it (GB), phone/fax: +39089233463(SDM); +39089969741/+39089969602 (GB)

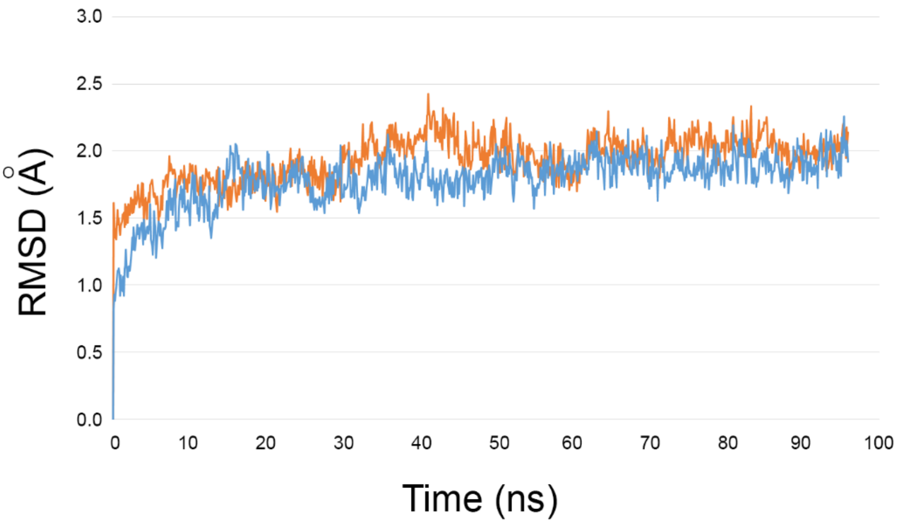

Figure S1. RMSD (Å) plots for Model H computed from molecular dynamics simulations (simulation time = 100 ns). Averaged RSMD = 0.183 Å.

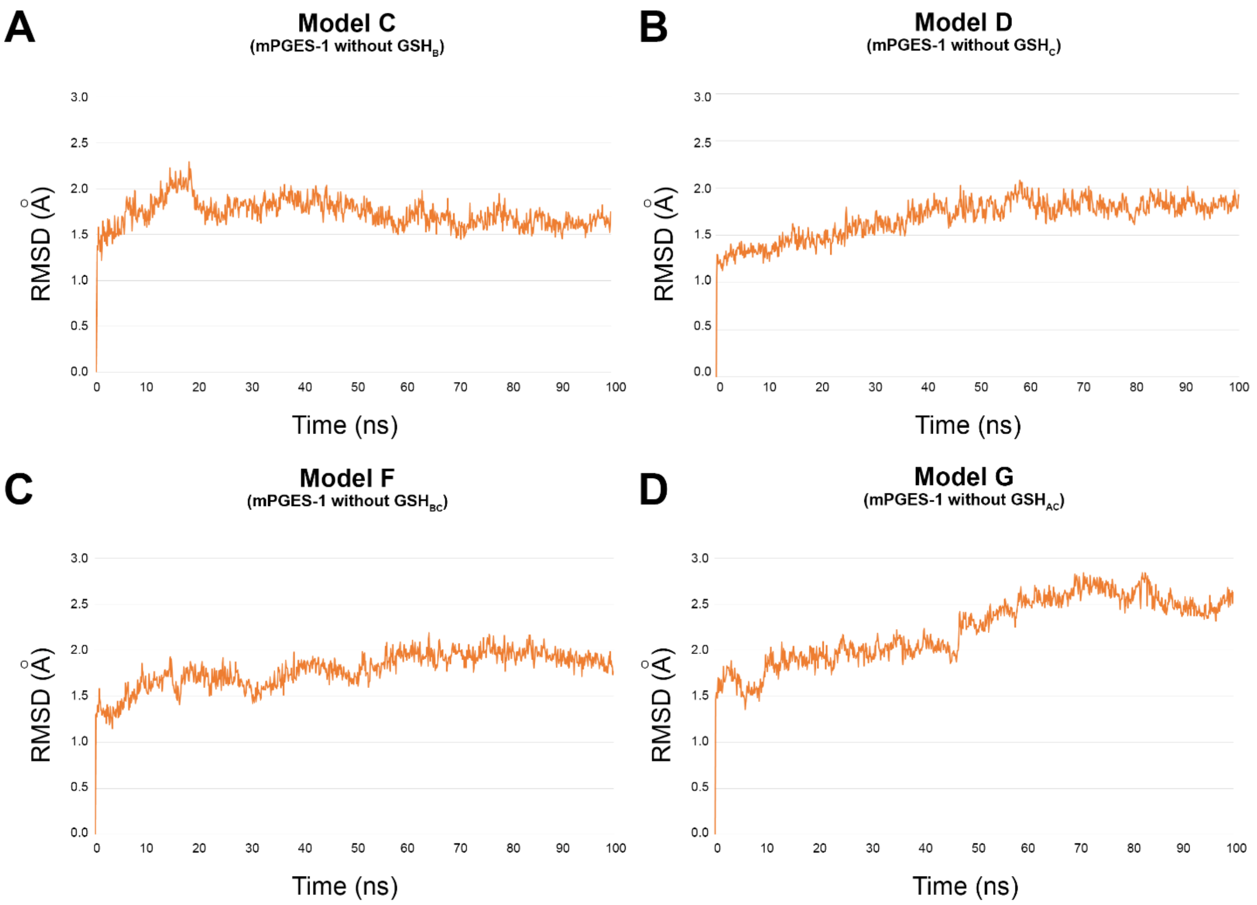

Figure S2. RMSD (Å) plots for Model C (panel A), D (panel B), F (panel C), and G (panel D) systems, related to mPGES-1 enzyme differently bound to GSH molecules (see Table 1), computed from molecular dynamics simulations (simulation time = 100 ns).

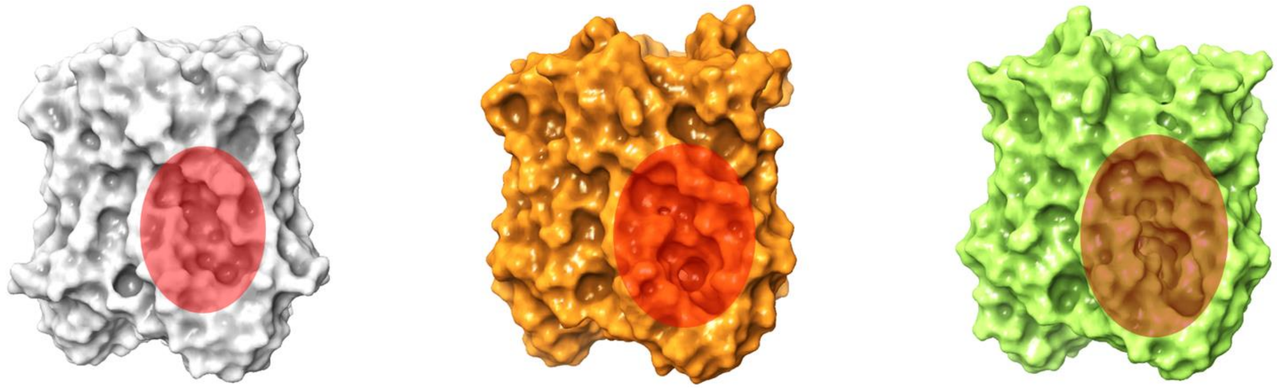

Figure S3. Molecular surface representations highlighting the different structural changes in the ligand binding sites of mPGES-1 (highlighted with transparent red ovals) for Model A (left), Model E (center), and Model H (right) at the end of the related molecular dynamics simulations (100 ns).

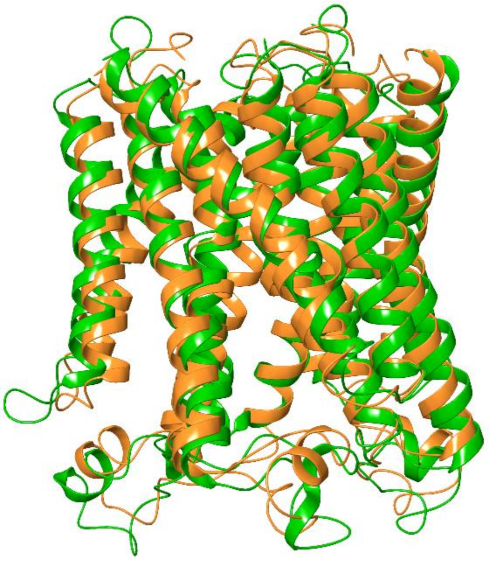

Figure S4. Superposition between mPGES-1 crystal structure (PDB code: 4AL0) (represented in orange ribbons) and the related three-dimensional structure obtained after molecular dynamics simulation at 5.1 μs for A) model A (mPGES-1 structure represented in green ribbons).

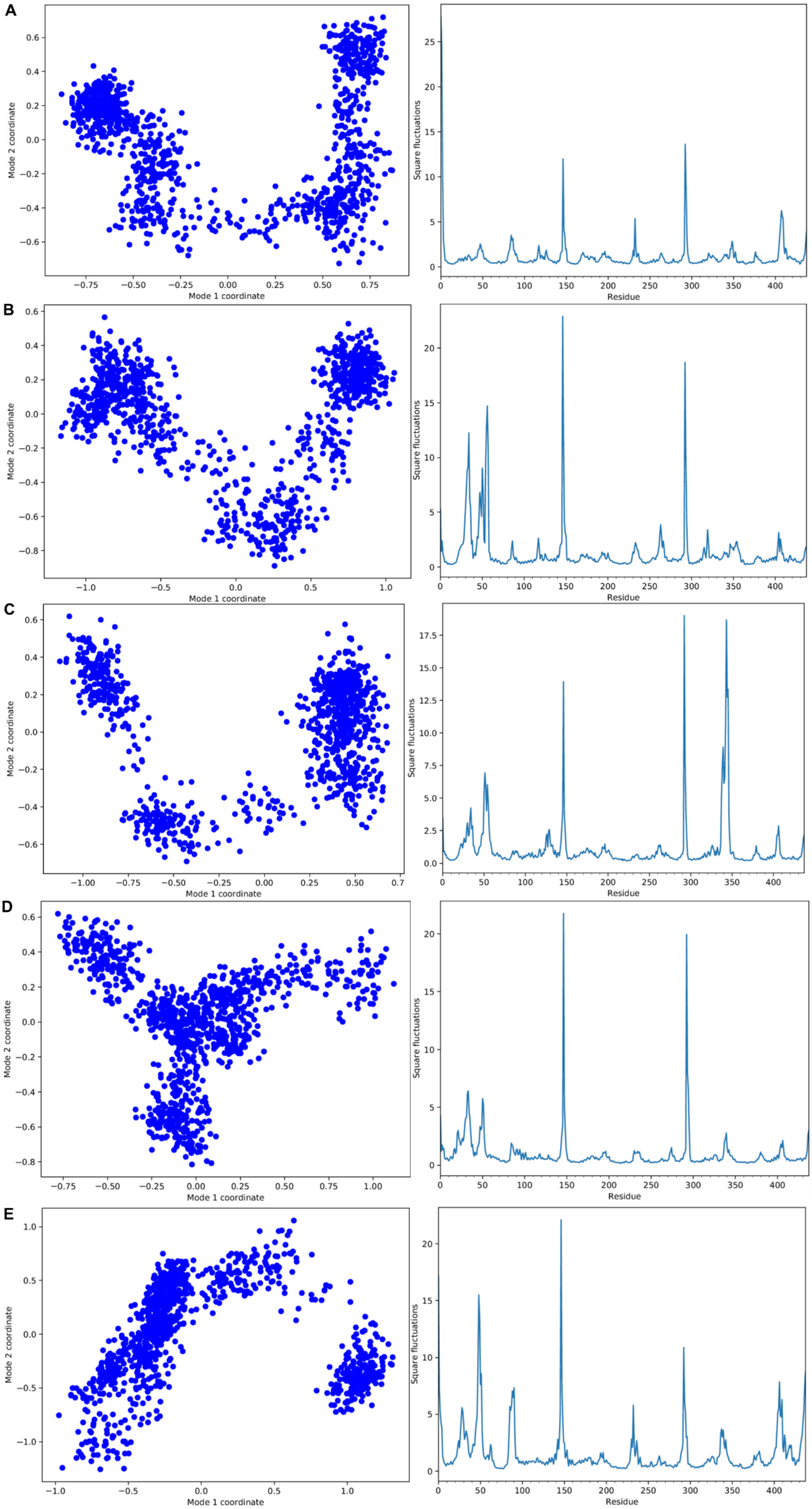

Figure S5. Essential dynamics analysis of microsecond-scale simulation (10 μs), arbitrarily divided in five time blocks: A, 0.721 μs; B, 1.122 μs; C, 1.354 μs; D, 1.893 μs; E, 4.909 μs. Left panels: projection of protein conformations onto PC1 and PC2. Right panels: Structural variation along PC1.

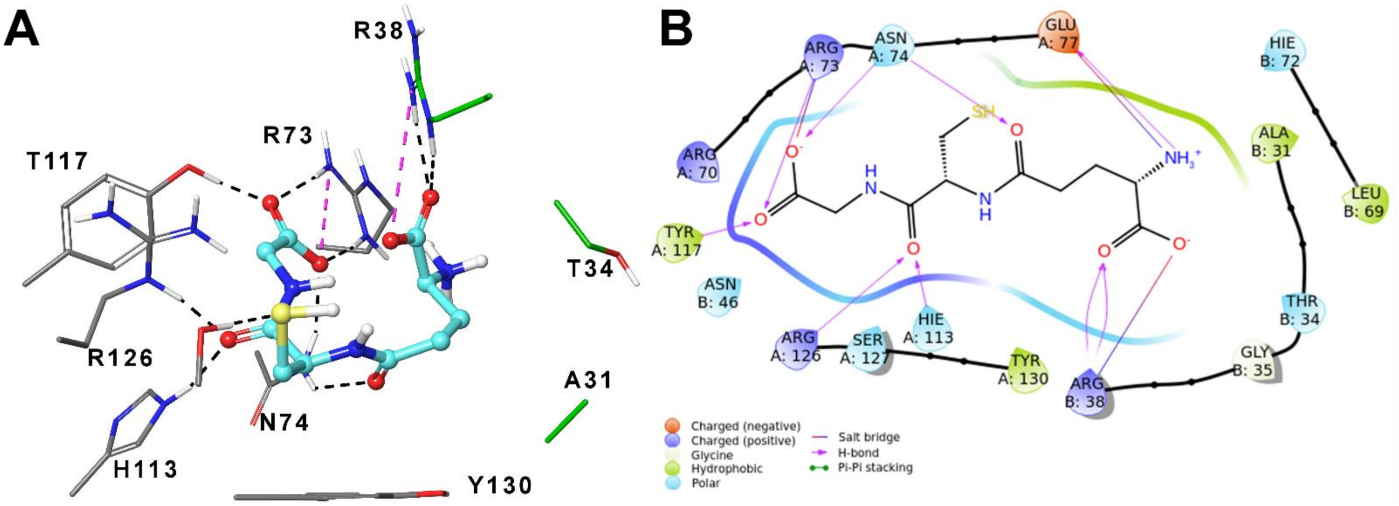

Figure S6. A) Three-dimensional model of the interactions given by GSH with mPGES-1, representative of three subunits. The GSH and protein are respectively depicted by stick-and-balls and tube (coloured: C, cyan for GSH and grey for protein; polar H, white; N, dark-blue; O, red; S, yellow). The dashed black and violet lines respectively indicate the hydrogen bonds and ionic interaction between GSH and protein. B) 2D panel interactions formed by GSH with mPGES-1. The molecular structure of GSH is depicted in black, while the protein amino acids are indicated with three letter code, encircled with coloured lines (negatively charged amino acids, red; positively charged amino acids, dark blue; polar amino acids, light blue; hydrophobic amino acids, green circles).

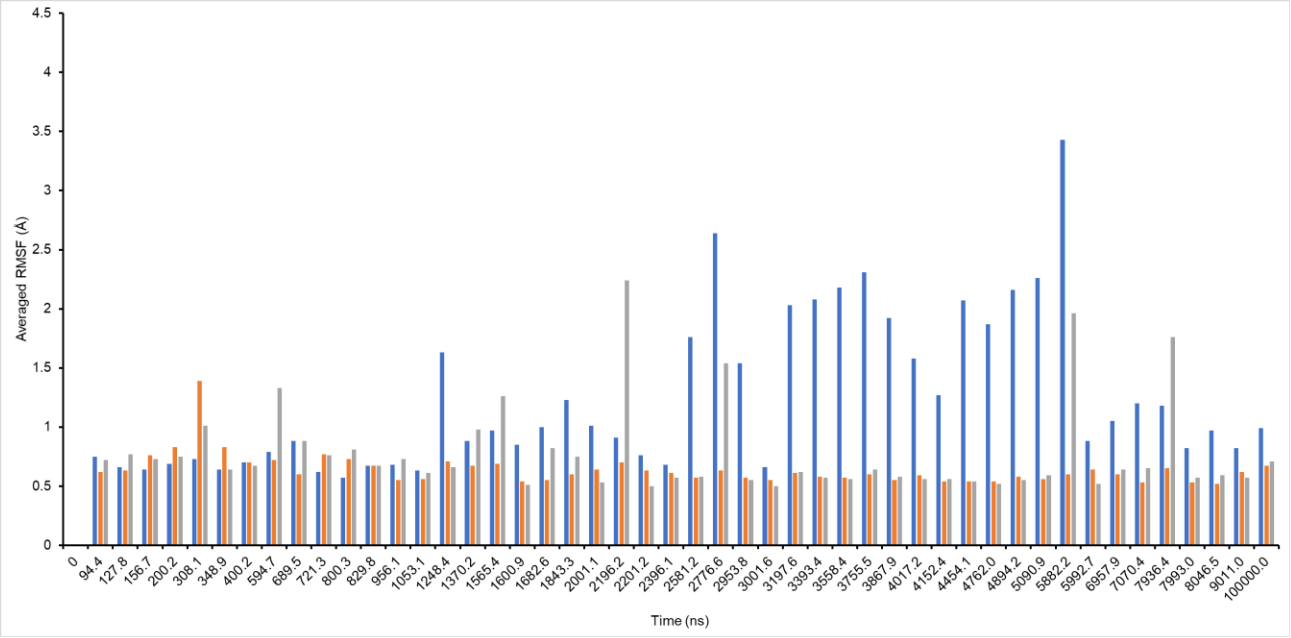

Figure S7. The Root Mean Square Fluctuation (RMSF) of R73 side chain as function of simulation time (ns). The blue, orange and green bars refer to chains A, B and C, respectively.

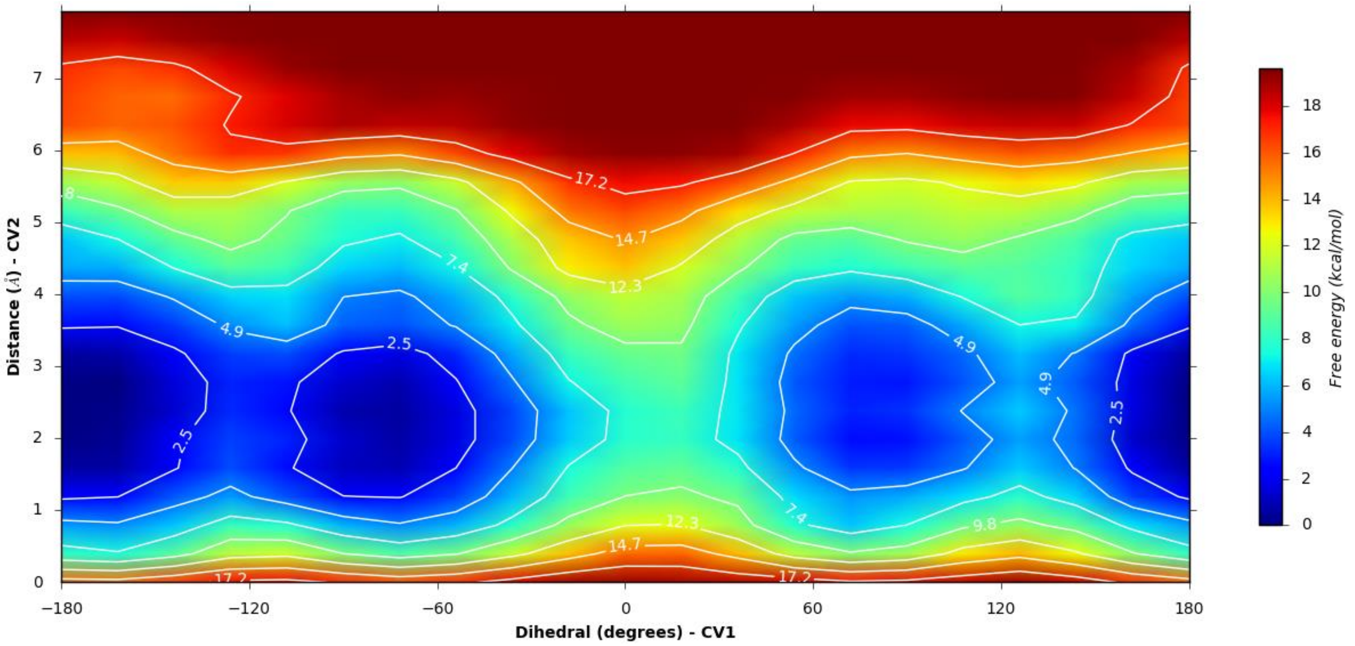

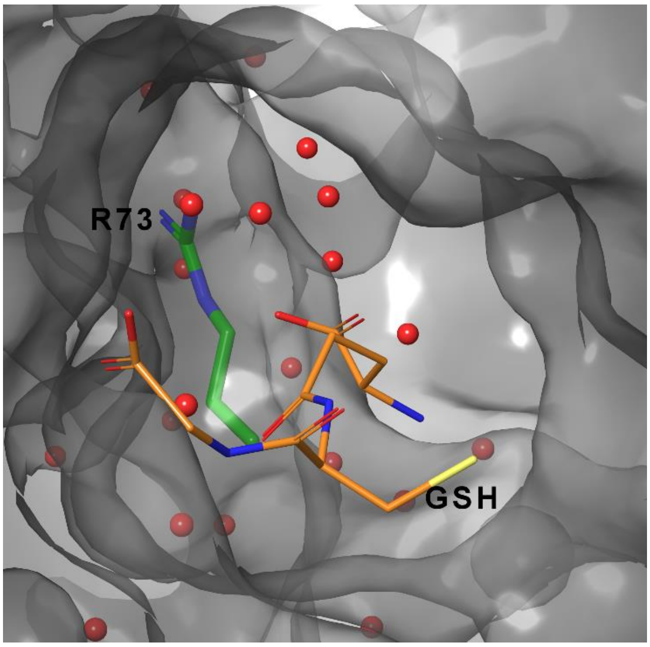

Figure S8. (top) The free energy contour plot from metadynamics. (bottom) The conformational arrangement of R73 at last frame of metadynamics simulation (50 ns). The protein is represented by grey molecular surface, whereas R73 and GSH by green and orange tubes, respectively, with the following atom colour code: C, as for tubes; O, red; N, blue; S yellow. The water molecules are depicted as red balls.

Table S1. Average area per lipid headgroup (APL) [Å^2].

	APL_{top} (Å^2)	APL_{bottom} (Å^2)	D_{HH} (nm)
Model A	71,2	70,3	3,7
Model B	75,5	70,6	3,5
Model C	77,0	71,3	4,0
Model D	76,9	71,3	4,0
Model E	76,5	71,6	3,5
Model F	76,2	73,1	4,0
Model G	77,1	70,0	4,1
Model H	72,2	66,4	3,5

Table S2. Predicted ΔG_{bind} (± SEM, kcal/mol) for alanine scanning of binding site residues by using MM-GBSA of Schrödinger.

residue	GSH
A31	-
T34A	-1.22 ± 0.03
G35	-
R38A	-0.17 ± 0.01
N46A	-0.03 ± 0.01
L69A	-0.13 ± 0.01
R70A	-0.35 ± 0.01
H72A	-0.03 ± 0.00
R73A	-0.35 ± 0.02
N74A	-1.06 ± 0.01
E77A	-0.30 ± 0.01
H113A	-0.09 ± 0.01
Y117A	-0.11 ± 0.00
R126A	-0.27 ± 0.05
S127A	-0.64 ± 0.02
Y130A	-0.92 ± 0.02
